# A Cuproptosis‐Related lncRNA Signature Predicts Prognosis and Shapes the Immune Landscape in Primary Lower‐Grade Glioma

**DOI:** 10.1155/genr/3061843

**Published:** 2025-12-08

**Authors:** Mengyang Wang, Jianmei Yang, Lei Shen, Jingyi Yang, Ming Luo, Faliang Duan

**Affiliations:** ^1^ Department of Neurosurgery, Wuhan No. 1 Hospital, Wuhan, 430022, Hubei, China, whyyy.com; ^2^ Department of Gastroenterology, Hubei Provincial Hospital of Integrated Chinese & Western Medicine, Wuhan, 430015, Hubei, China; ^3^ Department of Neurosurgery, Zhongnan Hospital of Wuhan University, Wuhan, 430071, Hubei, China, znhospital.cn

**Keywords:** cuproptosis, immune infiltration, immune microenvironment, lncRNA, primary lower-grade glioma

## Abstract

Glioma represents the most prevalent intracranial neoplasms. Lower‐grade gliomas (LGGs) are an important subtype of glioma, but the risk stratification of LGG has not been fully elucidated. As a recently recognized form of programmed cell death, cuproptosis is intimately tied to mitochondrial metabolism. Moreover, investigations have revealed that cuproptosis has been implicated in tumor initiation and progression. Long noncoding RNAs (lncRNAs) are engaged in diverse biological processes and connected with the malignant phenotype of gliomas. However, the significance of cuproptosis‐related lncRNAs (CRLs) in LGG development remains not fully elucidated. In this work, 963 CRLs were identified using correlation analysis, and a prognostic signature was constructed based on LASSO and multivariate Cox regression analyses. This signature comprised four CRLs: *AC002456.1*, tumor protein p63 regulated 1‐antisense RNA 1 (*TPRG1-AS1*), *AC098851.1*, and LYR motif containing 4‐antisense RNA 1 (*LYRM4-AS1*). According to the CRL‐based signature, LGG patients were classified into distinct risk groups. To investigate the involvement of biological processes in each LGG sample, we performed gene set variation analysis (GSVA) and gene set enrichment analysis (GSEA) comparing the different risk stratifications. Subsequently, the Estimation of STromal and Immune cells in MAlignant Tumor tissues using Expression (ESTIMATE) data and the tumor immune dysfunction and exclusion (TIDE) were utilized to access the tumor immune landscape of LGG samples. The results demonstrated that the immune landscapes of different risk stratifications differed significantly. Furthermore, we explored the association between the CRL risk signature and immunotherapy effectiveness using the IMvigor210 dataset. Several prospective drugs targeting samples with high scores were predicted, namely, MG‐132, PLX‐4720, AZD6482, and BMS‐536924. We verified the antiglioma effect of MG‐132 in vitro. Moreover, experiments conducted in vitro demonstrated that knockdown of the expression of the CRLs *TPRG1-AS1* and *LYRM4-AS1* might impair the migration and proliferation capacity of glioma cells. Taken together, these results indicate that CRLs are linked to prognosis and immune characteristics in LGG and give innovative therapeutic methods for individuals with LGG across different risk stratifications.

## 1. Introduction

Glioma represents the most common primary intracranial neoplasms [1]. Following the World Health Organization (WHO) classification system, gliomas are categorized into 4 grades, and the increasing grade denotes rising degrees of aggressiveness. Grades II and III gliomas are collectively referred to as lower‐grade gliomas (LGGs) [2]. In spite of tremendous advances in the discovery of anticancer drugs, effective drugs to treat LGG remain scarce, and patients with LGG have a grim prognosis [3]. Recent advances have introduced targeted therapies such as Vorasidenib, which inhibits mutant IDH1/2 and has shown promising efficacy in non‐enhancing LGG, representing a significant breakthrough in targeting this prevalent molecular alteration [4, 5]. Hence, there is an urgent demand for novel biomarkers to forecast the outcome of individuals with LGG and discover prospective therapeutic strategies focusing on LGG [6].

Long noncoding RNAs (lncRNA) participate in various biological processes, such as regulating gene transcription and post‐transcriptional expression, thereby promoting the development of tumors [7]. LncRNAs have also been implicated in regulating tumor metabolism, particularly glycolysis, which is closely associated with glioma progression and metastasis [8, 9]. Moreover, previous studies have linked lncRNAs to the malignant phenotype of gliomas [10]. Research indicated that hypoxia‐related lncRNAs might be new indicators to forecast the outcome of individuals with LGG and prospective candidates for LGG therapy [11]. Another study established a pro‐neural to mesenchymal transition‐related lncRNA signature to predict glioma survival [12]. Xu et al. found that *HOXA11-AS* affects glioma’s malignant phenotype via the *miR-130a-5p-HMGB2* axis [13]; another study suggested that lncRNA *LINC01857* promotes glioma development by regulating the *miR-1281/TRIM65* axis [14].

Previous research has revealed that the dysregulation of programmed cell death induced by lncRNA, such as pyroptosis and ferroptosis, is involved in the gliomas’ occurrence and development. Other studies have emphasized the role of oxidative stress and apoptosis resistance in glioma development, suggesting that redox imbalance and related programmed cell death mechanisms could be therapeutic vulnerabilities [15, 16]. For example, lncRNA *TMEM161B-AS1* can promote ferroptosis in glioma cells by targeting *hsa-miR-27a-3p* and inhibiting their proliferation and invasion [17], and lncRNA *PCED1B-AS1* can inhibit apoptosis and promote proliferation in glioma cells by regulating *miR-194-5p/PCED1B* [18]. Tsvetkov et al. has described cuproptosis as a unique programmed cell death process that may greatly affect the malignant phenotype of glioma [19]. Unlike apoptosis or ferroptosis, cuproptosis occurs when excess intracellular copper directly binds to lipoylated components of the tricarboxylic acid cycle, causing protein aggregation and destabilization of iron–sulfur cluster proteins [20]. This results in proteotoxic stress and ultimately causes cell death in a mitochondrial respiration‐dependent manner. Previous research has established a cuproptosis‐related lncRNAs (CRL‐derived) prognostic signature capable of estimating survival in glioma patients [21]. Moreover, another study first studied the relationship between CRLs and LGG [22].

Nevertheless, no study has altogether expounded the role of CRLs in primary LGG. RNA‐sequencing (RNA‐seq) and clinical information of primary LGG patients were obtained from The Cancer Genome Atlas (TCGA) database to identify potential CRLs and develop a novel CRL‐based prognostic signature. These data were used to analyze their relationship with the immune landscape of primary LGG and the efficacy of immunotherapy and chemotherapy by predicting potential medications.

## 2. Materials and Methods

### 2.1. Data Collection

**Figure 1:**
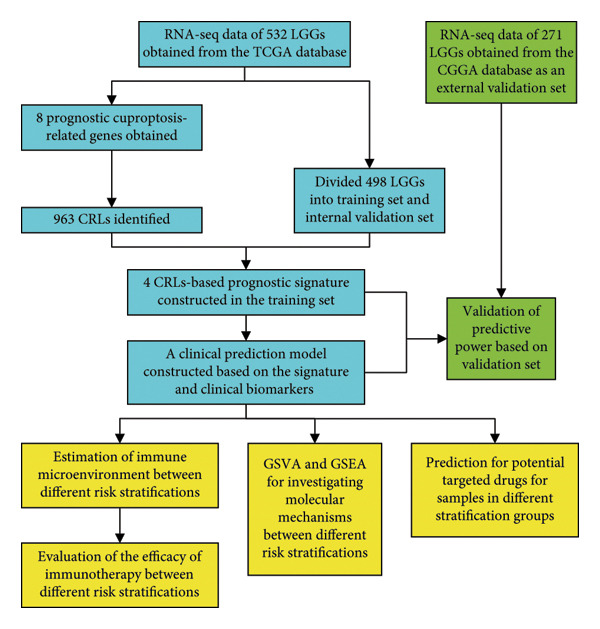
Flow diagram. LGG, lower‐grade glioma. CRL, cuproptosis‐related lncRNAs. GSVA, gene set variation analysis. GSEA, gene set enrichment analysis. RNA‐seq, RNA‐sequencing.

Figure 1 displays the process chart for the current study. A total of 498 individuals with primary LGG were comprised in this research, while samples labeled as “01B” and “02” in the TCGA barcode were excluded. Gene expression profiles and clinical data of patients with primary LGG were obtained from the GDC database. 498 samples were randomly divided into a training (*n* = 348) and an internal validation set (*n* = 150) using the R package “caTools” (https://cran.r-project.org/web/packages/caTools/). Meanwhile, RNA‐seq data and clinical data of 271 patients with primary LGG in the Chinese Glioma Genome Atlas (CGGA) were obtained as the external validation set [23]. The characteristics of patients with LGG are reported in Table 1, and there were no significant variations in the clinical features between the training and internal validation sets. In addition, to investigate the predictive validity of the CRL signature for immunotherapy effectiveness, we extracted a set of IMvigor210 datasets on atezolizumab for the treatment of urothelial carcinoma [24].

**Table 1:** Clinical characteristics of patients in the dataset.

		TCGA‐training set (*n* = 348)	TCGA‐validation set (*n* = 150)	CGGA‐validation set (*n* = 271)	*p* value
Age	> 45	130	69	70	0.239
	≤ 45	218	82	201	

Gender	Male	192	85	151	0.769
	Female	156	65	120	

Histologic grade	WHO II	174	66	131	0.241
	WHO III	174	84	142	

*IDH* mutation	Mutant	286	113	177	0.201
	Wild type	58	32	66	
	NA	4	5	31	

1p/19q Codel^∗^	Codel	113	48	81	0.999
	Noncodel	231	97	158	
	NA	4	5	32	

*Note:* *p* value, significant difference between the TCGA training set and the TCGA internal validation set. IDH, isocitrate dehydrogenase.

Abbreviations: NA = not available, WHO = World Health Organization.

^∗^1p/19q codeletion status.

### 2.2. Identification of CRLs

Based on GEPIA2 (https://gepia2.cancer-pku.cn/#index), univariate Cox analysis was conducted to discover cuproptosis‐related genes linked with LGG prognosis [25]. These included *CDKN2A*, *DLAT*, *DLD*, *FDX1*, *GLS*, *LIAS*, *LIPT1*, *MTF1*, *PDHA1*, *PDHB*, *SLC31A1*, *DBT*, *GCSH*, *ATP7A*, *ATP7B*, and *DLST*. Genes with log rank *p* < 0.05 were considered prognostic genes for LGG. Eight cuproptosis‐related genes associated with LGG prognosis, *GCSH*, *ATP7B*, *FDX1*, *GLS*, *DLAT*, *MTF1*, *ATP7A*, and *SLC31A1*, were selected to identify CRLs. Spearman correlation analysis was conducted for all lncRNAs in the RNA‐seq dataset. LncRNAs with |R| > 0.5 and *p* < 0.05, were recognized as CRLs and included in subsequent analyses.

### 2.3. Construction of Risk Signature and Clinical Prediction Models

Based on the identified CRLs, least absolute shrinkage and selection operator (LASSO) analysis and multivariate Cox regression were utilized to generate the risk signatures. The risk scores were calculated using the following algorithm: Risk Score = coefficient of lncRNA1 ∗ explncRNA1 + coefficient of lncRNA2 ∗ explncRNA2 + … + coefficient of lncRNAn ∗ explncRNAn; decreasing risk scores reflect decreasing risk of death. In this model, the coefficient represented the multivariate Cox regression coefficient of each lncRNA, while “exp” denoted its expression level. To explore the association between the risk score and clinical features of individuals with LGG, multivariate Cox regression analysis was utilized for screening independent risk variables affecting the prognosis of patients with LGG, and a clinical prediction model including the risk score and clinical features of individuals with LGG was constructed. For the construction and visualization of the nomogram and calibration curves, only samples with complete IDH mutation and 1p/19q codeletion information were included to ensure reliability. R packages “rms” (https://cran.r-project.org/web/packages/rms/) and “regplot” (https://cran.r-project.org/web/packages/regplot/) were used to generate and visualize the nomogram and calibration curves. The accuracy of prediction of the model was examined using receiver operating characteristic (ROC) curves.

### 2.4. Estimation of Immune Microenvironment

The Estimation of STromal and Immune cells in MAlignant Tumor tissues using Expression (ESTIMATE) data analysis was undertaken to evaluate the tumor purity of each sample using the “estimate” R package [26]. The infiltration of immunocytes was calculated using CIBERSORT [27]. The tumor immune dysfunction and exclusion (TIDE) was utilized to determine the effectiveness of immune checkpoint inhibitors (ICIs) in patients with LGG by defining malfunctioning T cells and infiltrating cytotoxic T‐lymphocyte levels [28]. Normalized TPM expression data were uploaded to the TIDE online platform (https://tide.dfci.harvard.edu). All other parameters use default settings.

### 2.5. Molecular Mechanism Exploration

To minimize false positives, the Wilcoxon rank‐sum test was applied to determine differential gene expression between the two groups. The fold changes of genes were calculated for gene set enrichment analysis (GSEA) [29]. Furthermore, gene set variation analysis (GSVA) was performed to assess the individual LGG samples based on the Gene Ontology (GO) terms and Kyoto Encyclopedia of Genes and Genomes (KEGG) pathways [30–32].

### 2.6. Potential Sensitive Drug Prediction

The drug‐sensitive data and expression data were collected from the Genomics of Drug Sensitivity in Cancer database and the Cancer Therapeutics Response Portal [33, 34]. Cells’ sensitivity to pharmaceuticals was calculated as a sensitivity score, and a lower sensitivity score signified more sensitivity to prospective medications. The R package “oncoPredict” was used to predict the sensitivity score of each sample in the current study [35].

### 2.7. Cell Culture and Transfection

The glioma cell lines, including U251 and T98G, acquired from the Cell Library of the Chinese Academy of Sciences (Shanghai, China), were cultured in complete medium (Dulbecco’s modified Eagle medium [DMEM, Servicebio] with 10% fetal bovine serum [FBS, Gibco] and 10 μl/mL penicillin–streptomycin [Biosharp]) in a humidified chamber at 37°C with 5% CO2. For the drug‐sensitivity experiments, glioma cells were grown in normal complete medium and complete medium with 20 μM MG‐132 (MedChemExpress, USA), respectively.

The sense sequence of small interfering (si) RNA against human *LYRM4-AS1* (5′‐GCU​CUC​AUU​CAG​CAU​UUA​UTT‐3′), against human *TPRG1-AS1* (5′‐ACA​UUA​AAU​UGG​AGG​ACU​ATT‐3′), a negative control siRNA (5′‐UUC​UCC​GAA​CGU​GUC​ACG​UTT‐3′), and RNA TransMate (E607402) were acquired from Sangon Biotech (Shanghai, China). The transfection was conducted pursuant to the manual. Firstly, 3 × 10^5 glioma cells were seeded in each well of the 6‐well plate and cultured in complete medium overnight. After overnight, the culture medium was replaced by DMEM with 10 nM siRNA, 0.6% RNA TransMate, and no FBS for 8 h for the transfection. Then the culture medium was changed with complete medium after 8 h of transfection. Finally, the glioma cells transfected with siRNAs were cultured for another 24 h, and the transfection was considered completed. After 24 h of transfection, the cells were employed for subsequent phenotypic experiments. The phenotypic experiments, including colony formation assay, cell counting Kit‐8 proliferation assay, and wound healing assay, were conducted as reported in the preceding study [36, 37].

### 2.8. RNA Extraction, cDNA Synthesis, and Quantitative Real‐Time PCR (qPCR)

Total RNA from glioma cells was extracted using Takara RNAiso Plus (Takara Bio. Inc., Otsu, Shiga, Japan) following the manufacturer’s protocol. The detailed extraction and qPCR procedures have been described previously in our publication [37] and were followed without modification. Briefly, cDNA synthesis was performed using the HiScript II Q RT SuperMix for qPCR Kit (Vazyme Medical Technology, Nanjing, China), and relative gene expression levels were calculated using the 2^–ΔΔCt^ method as reported in our earlier study. Primer sequences for qPCR were listed below: *GAPDH*‐F, 5′‐GGA​GCG​AGA​TCC​CTC​CAA​AAT‐3′, *GAPDH*‐R, 5′‐GGC​TGT​TGT​CAT​ACT​TCT​CAT​GG‐3′; *LYRM4-AS1*‐F, 5′‐ATA​AAG​AAG​GTC​CGG​CAA​G‐3′, *LYRM4-AS1*‐R, 5′‐TGC​CTC​TCA​TCA​TCC​CA‐3′; *TPRG1-AS1*‐F, 5′‐ATG​CAG​GCG​AAA​GAG​GT‐3′, *TPRG1-AS1*‐R, 5′‐CAC​GCA​CAC​TTA​CTG​ACG​A‐3′.

### 2.9. Statistical Analysis

Statistical analyses and visualizations were performed using R Version 4.1.3. Gene expression correlations were determined using Spearman correlation analysis. The Student’s *t*‐test was used to compare the differences between the two groups. The Wilcoxon test was performed to identify differentially expressed genes. The chi‐square test was employed to examine if the clinical features of the training and validation sets were associated. The predictive accuracy of the risk scores was examined using a time‐dependent ROC curve analysis. Two‐sided *p* ≤ 0.05 (^∗^) was regarded as statistically significant and was further stratified to *p* < 0.01 (^∗∗^), *p* < 0.001 (^∗∗∗^), and *p* < 0.0001 (^∗∗∗∗^).

## 3. Results

### 3.1. Identification of Prognostic Cuproptosis‐Related Genes and Corresponding CRLs

Univariate Cox regression analysis identified eight cuproptosis‐related genes significantly linked to the prognosis of LGG (Figures 2(a), 2(b), 2(c), 2(d), 2(e), 2(f), 2(g), 2(h)). Among the eight cuproptosis‐related genes, the hazard ratio (HR) of only two genes, *GCSH* (*p* = 0.0068) and *ATP7B* (*p* = 0.03) were considered as the protective factors. The HR of the other six genes, including *FDX1* (*p* = 0.00013), *GLS* (*p* = 0.0058), *DLAT* (*p* = 0.0068), *MTF1* (*p* = 0.0044), *ATP7A* (*p* = 0.0024), and *SLC31A1* (*p* = 0.00017) were considered as risk factors.

Figure 2KM curve of eight cuproptosis‐related genes associated with LGG prognosis. (a) Adrenal ferredoxin (FDX1). (b) Glutaminase (GLS). (c) Dihydrolipoyl transacetylase (DLAT). (d) Metal regulatory transcription factor 1 (MTF1). (e) ATPase copper transporting beta (ATP7A). (f) High affinity copper uptake protein 1 (SLC31A1). (g) Glycine cleavage system protein H (GCSH). (h) ATPase copper transporting beta (ATP7B). (i) Eight cuproptosis‐related genes are associated with LGG prognosis. KM, Kaplan–Meier. LGG, lower‐grade glioma.

**Figure:**
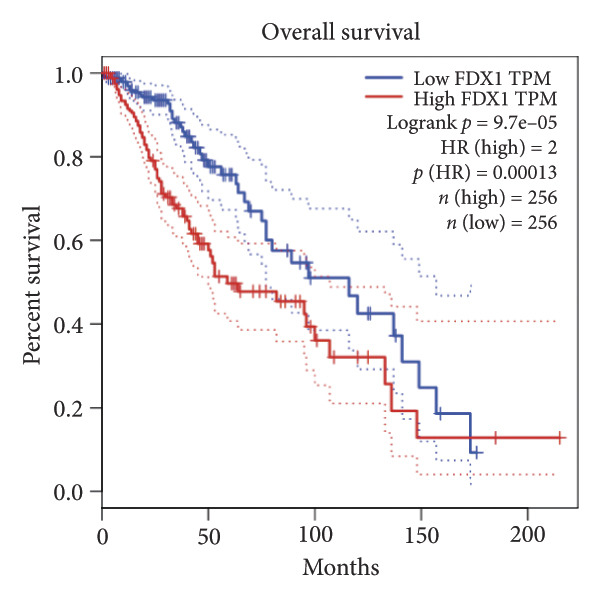
(a)

**Figure:**
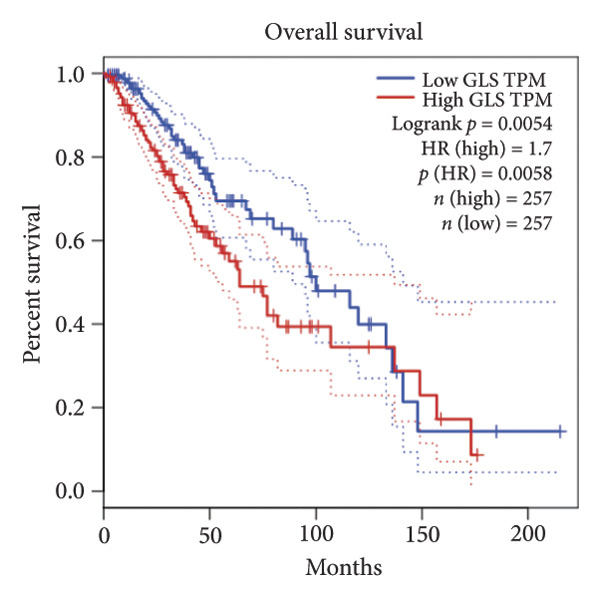
(b)

**Figure:**
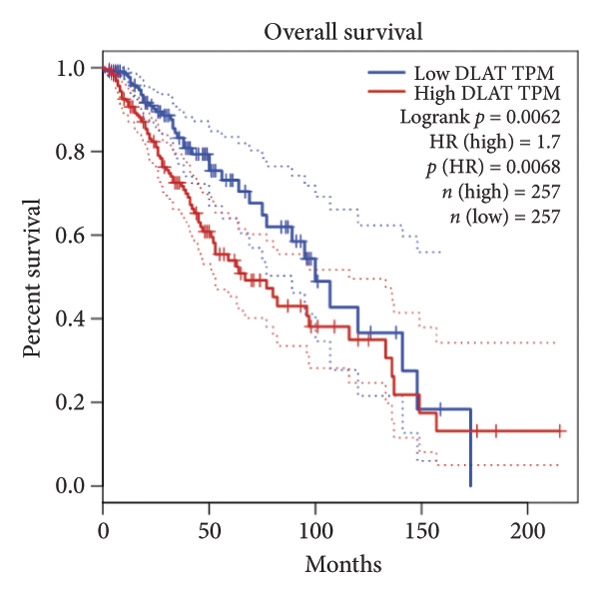
(c)

**Figure:**
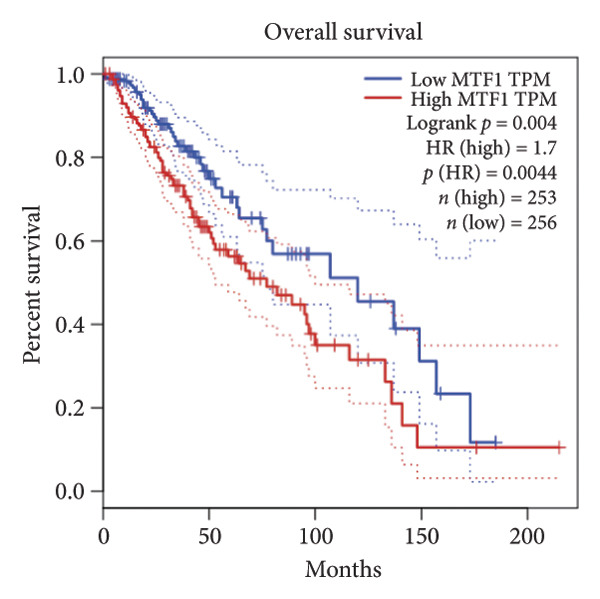
(d)

**Figure:**
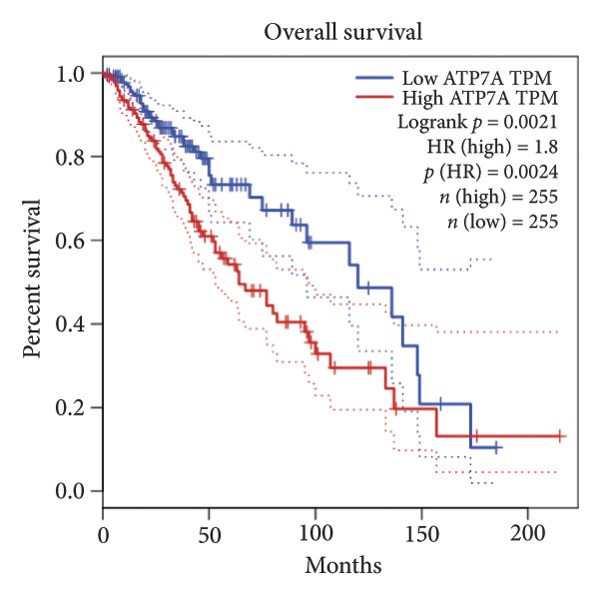
(e)

**Figure:**
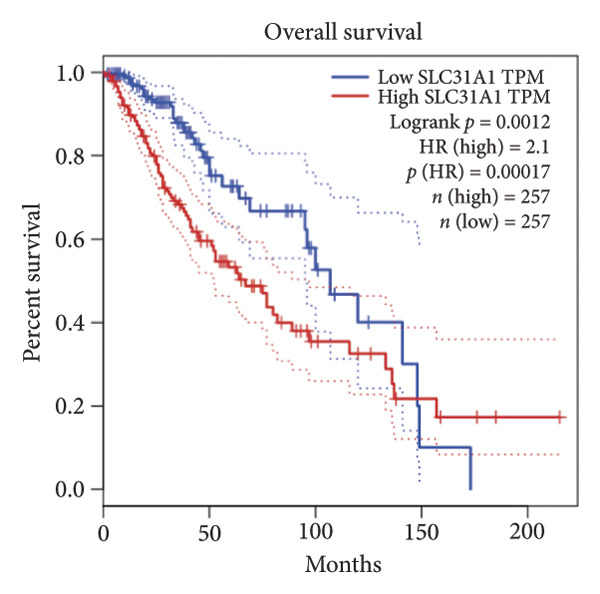
(f)

**Figure:**
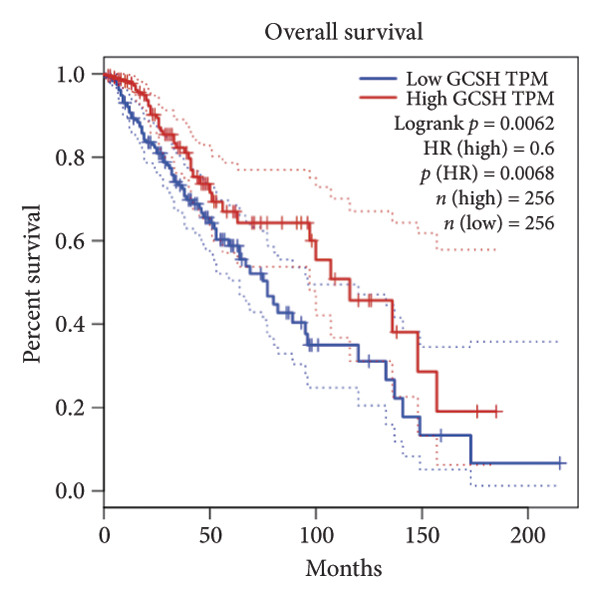
(g)

**Figure:**
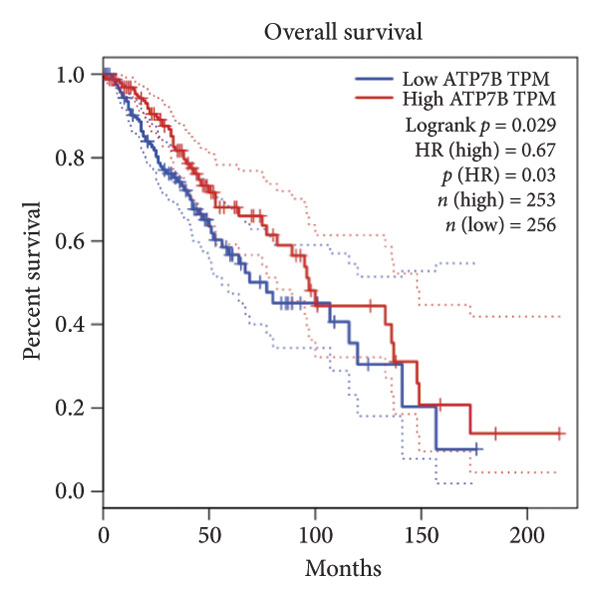
(h)

**Figure:**
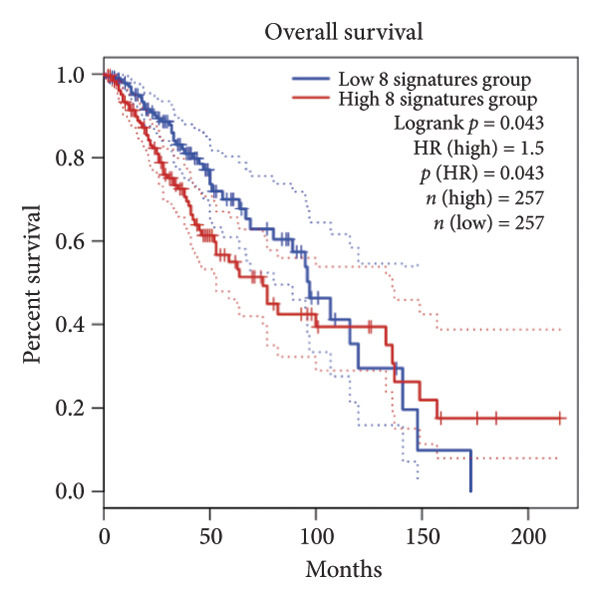
(i)

In addition, these eight cuproptosis‐related gene signatures showed a significant correlation with LGG prognosis (*p* = 0.043) (Figure 2(i)). Subsequently, 963 CRLs were identified using Spearman correlation analysis (Supporting 1).

### 3.2. Construction of the Four‐CRL Signature Connected to the Prognosis of Individuals With LGG

The TCGA dataset was divided into a training and an internal validation set randomly. LASSO regression analysis was performed on 963 CRLs, and four CRLs, *AC002456.1*, tumor protein p63 regulated 1‐antisense RNA 1 (*TPRG1-AS1*), *AC098851.1*, and LYR motif containing 4‐antisense RNA 1 (*LYRM4-AS1*), were chosen for further analysis (Figures 3(a) and 3(b)). Figure 3(c) shows the correlation between the four CRLs and the eight prognosis‐related cuproptosis‐related genes. According to the results, *AC002456.1* was correlated with *FDX1* (*R* = 0.52), *AC098851.1* was correlated with *MTF1* (*R* = 0.52), *TPRG1-AS1* was correlated with *MTF1* (*R* = 0.51), and *LYRM4-AS1* was correlated with *DLAT* (*R* = 0.52), *MTF1* (*R* = 0.53), *ATP7A* (*R* = 0.63), and *SLC31A1* (*R* = 0.71). Meanwhile, the results of univariate Cox analysis showed that *AC002456.1* (*p* < 0.001), *TPRG1-AS1* (*p* < 0.001), *AC098851.1* (*p* < 0.001), and LYRM4‐AS1 (*p* < 0.001) were linked with the prognosis of individuals with LGG in TCGA (Figure 3(d)) and CGGA (Figure 3(e)).

Figure 3Four CRLs identified by LASSO regression. (a and b) LASSO regression analysis to screen CRLs. (c) Heatmap showing the correlation between four CRLs and eight cuproptosis‐related genes. (d and e) Univariable Cox regression analysis forest plot of four CRLs in TCGA and CGGA. CRL, cuproptosis‐related lncRNAs. LASSO, least absolute shrinkage and selection operator.

**Figure:**
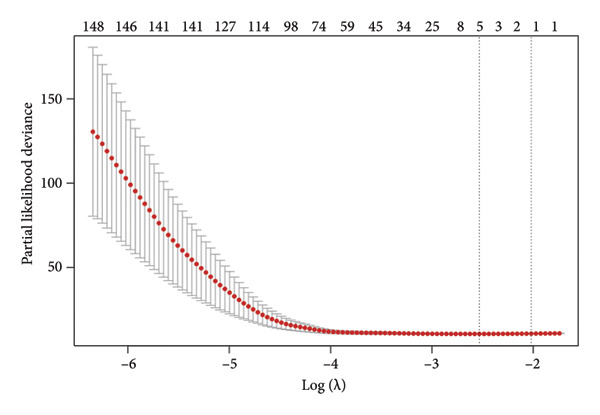
(a)

**Figure:**
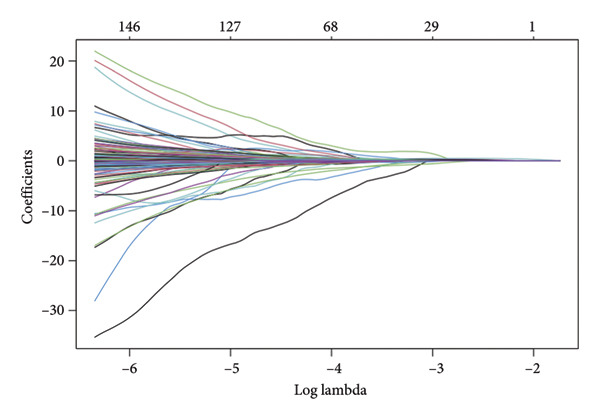
(b)

**Figure:**
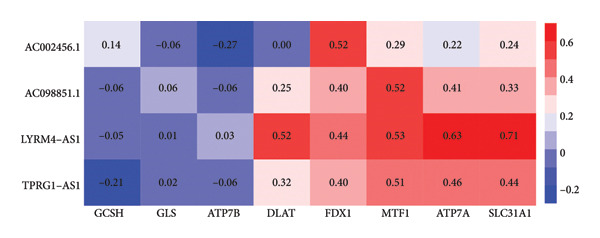
(c)

**Figure:**
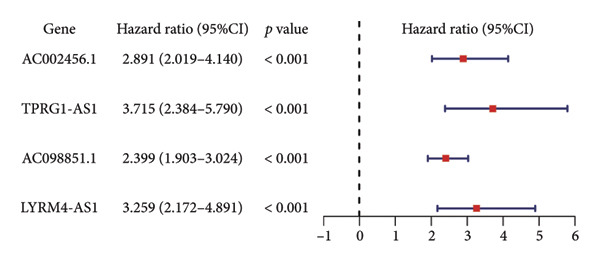
(d)

**Figure:**
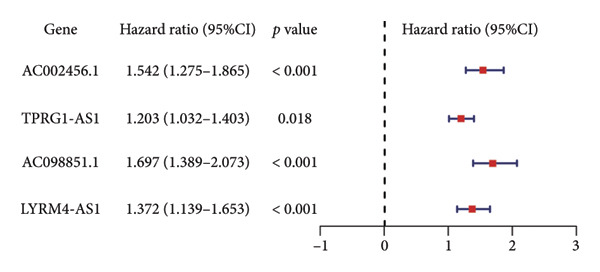
(e)

Subsequently, a prognostic signature including *AC002456.1*, *TPRG1-AS1*, *AC098851.1*, and *LYRM4-AS1* was constructed (Figure 4(a)). The four‐CRL signatures divided patients into high‐ and low‐risk stratifications, and principal component analysis revealed satisfactory separation between different risk stratifications (Figure 4(b)). In the training set, patients with relatively short survival times (*p* < 0.0001) and a censored status were enriched in the high‐risk stratification. The area under the curve (AUC) of the risk scores for predicting 1‐, 3‐, and 5‐year survival of patients with LGG was 0.822, 0.845, and 0.793, respectively (Figure 4(c)). Similar to the training set, individuals in the high‐risk stratification of the internal validation set had a shorter overall survival time and intense censored status (*p* < 0.0001), and the AUC of the risk score in predicting 1‐, 3‐, and 5‐year survival of patients with LGG was 0.848, 0.821, and 0.743, respectively, in the internal validation set (Figure 4(d)). To further validate the precision of our prognostic signature, the CGGA dataset was used as the external validation set, and the patients in the high‐risk stratification had a worse prognosis, with the AUC of the risk score in predicting 1‐, 3‐, and 5‐year survival was 0.523, 0.555, and 0.609, respectively (Figure 4(e)). Heatmaps showed that these four CRLs (*AC002456.1*, *TPRG1-AS1*, *AC098851.1*, and *LYRM4-AS1*) were enriched in individuals with higher risk scores in all datasets (Figures 4(c), 4(d), 4(e)).

Figure 4Construction of the four‐CRL signature. (a) Multivariate Cox regression analysis forest plot of CRLs selected by LASSO. (b) Principal component analysis results. (c) Performance of the CRL prognostic signature in the TCGA training set, including the KM plot, the scatter plot of the risk score and survival time of each patient, the time‐dependent ROC curve, and the heatmap showing the expression of the four CRLs. (d) Performance of the CRL prognostic signature in the TCGA‐validation set. (e) Performance of the CRL prognostic signature in the CGGA‐validation set. CRL, cuproptosis‐related lncRNAs. KM, Kaplan–Meier. LASSO, least absolute shrinkage and selection operator. ROC, receiver operating characteristic.

**Figure:**
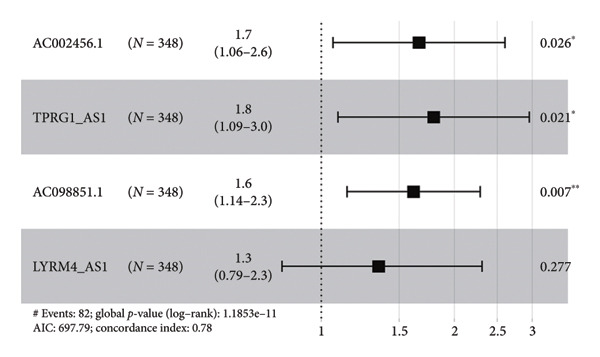
(a)

**Figure:**
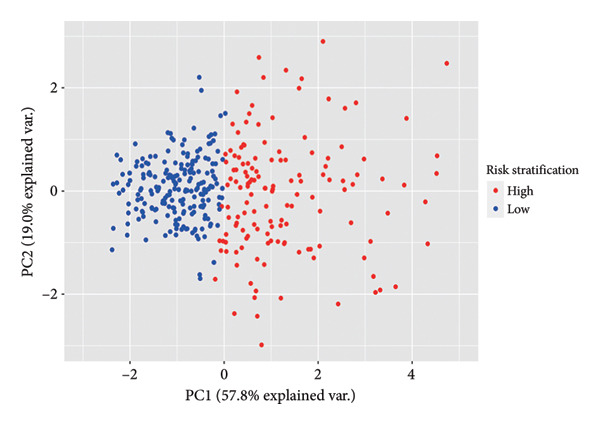
(b)

**Figure:**
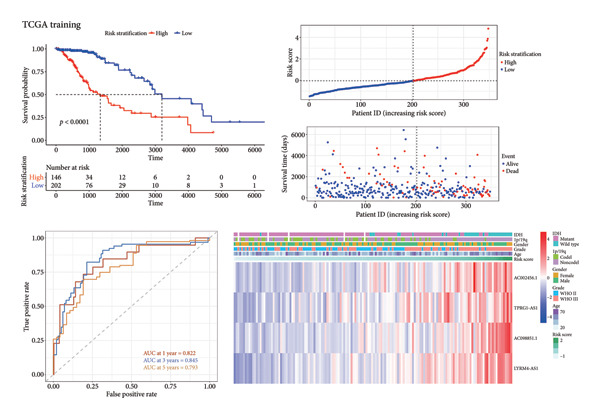
(c)

**Figure:**
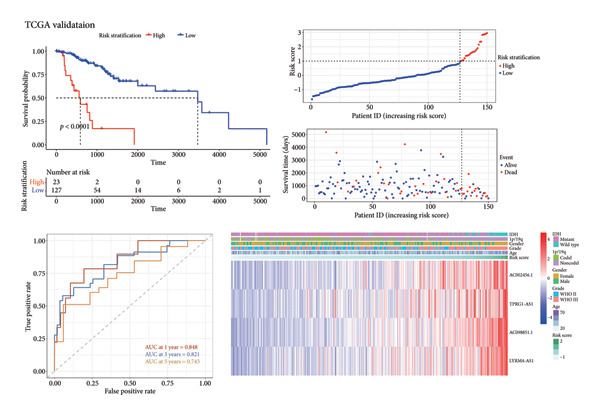
(d)

**Figure:**
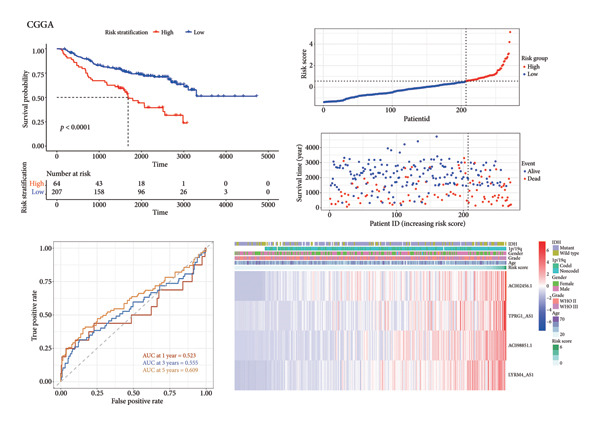
(e)

### 3.3. Construction of the Clinical Prediction Model Using the Four‐CRL Risk Signature and Clinical Features

As for several indicators of glioma, the risk score was considerably higher in individuals aged > 45 years (*p* < 0.001), WHO III (*p* < 0.0001), *IDH* wild type (*p* < 0.0001), and 1p/19q non‐codeleted (*p* < 0.0001) in the training set (Figure 5(a)). The connection between clinical features and the risk score revealed similar tendencies in the internal (Figure 5(b)) and external validation sets (Figure 5(c)). The nomogram model was generated based on the signature and clinical features, and the calibration plot showcased satisfactory consistency with the prediction of 3‐year and 5‐year overall survival, with the AUC for predicting the 1‐, 3‐, and 5‐year survival of patients with LGG was 0.867, 0.910, and 0.820, respectively (Figure 5(d)). To confirm the correctness of the nomogram, the similar nomogram models were constructed based on the internal validation set and the CGGA dataset, and the AUC was 0.910, 0.942 (Figure 5(e)), and 0.919 in the internal validation set and 0.739, 0.748, and 0.753, respectively, in the CGGA dataset (Figure 5(f)).

Figure 5Construction of a clinical prediction model. (a) Risk scores in different ages, grades, IDH status (c), and 1p/19q status of gliomas in the TCGA training set. (b) Risk scores in different ages, grades, IDH status (c), and 1p/19q status of gliomas in the TCGA validation set. (c) Risk scores in different ages, grades, IDH status (c), and 1p/19q status of gliomas in the CGGA‐validation set. (d) Performance of the clinical prediction model in the TCGA training set, including nomogram plot, time‐dependent ROC, and calibration plot. (e) Performance of the clinical prediction model in the TCGA validation set. (f) Performance of the clinical prediction model in the CGGA‐validation set. IDH, isocitrate dehydrogenase, ROC, receiver operating characteristic.

**Figure:**
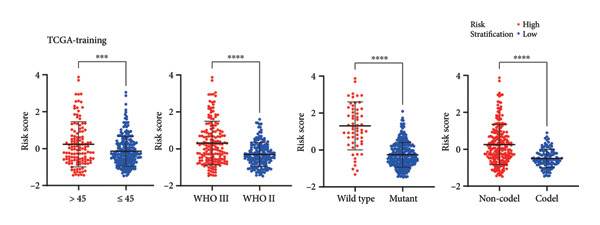
(a)

**Figure:**
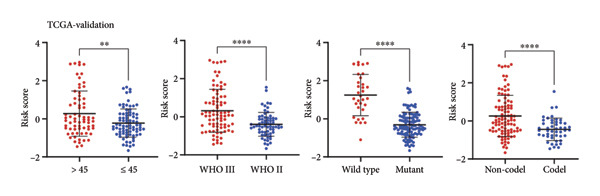
(b)

**Figure:**
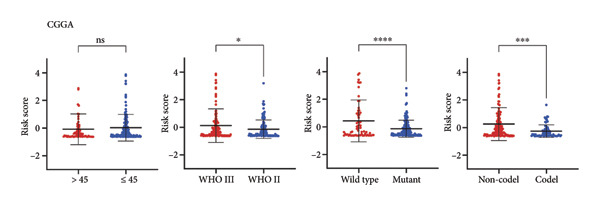
(c)

**Figure:**
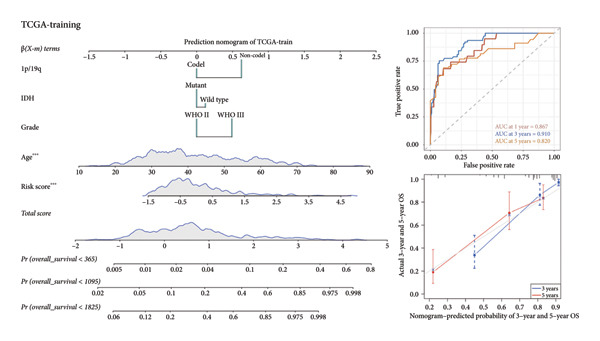
(d)

**Figure:**
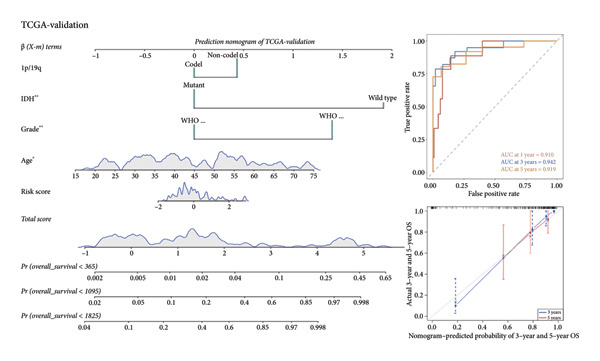
(e)

**Figure:**
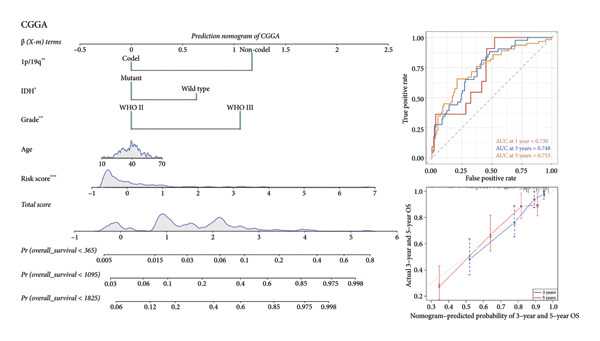
(f)

### 3.4. Different Immune Landscapes in Different Risk Stratifications

ESTIMATE analysis disclosed the correlations between the risk score and ESTIMATE score, higher the risk score, higher the immune (*p* = 1.1 × 10^ (−14)) and stromal scores (*p* = 9.4 × 10^ (−13)) and lower the tumor purity (*p* = 1.5 × 10^ (−15)) (Figure 6(a)). The samples in the CGGA dataset had the same trend (Figure 6(b)). CIBERSORT analyses indicated that the immune infiltration landscape was significantly different between the high‐ and low‐risk groups. Consistent with the TCGA dataset and CGGA dataset, samples in the high‐risk stratification group exhibited substantially greater infiltration of M2 macrophages (TCGA: *p* < 0.001, CGGA: *p* < 0.05), and reduced infiltration of activated natural killer (NK) cells (TCGA: *p* < 0.05, CGGA: *p* < 0.05) (Figures 6(c), 6(d)).

Figure 6Immune landscape of different risk stratifications. (a) ESTIMATE results of samples in the TCGA dataset. (b) ESTIMATE results of samples in the CGGA dataset. (c) Infiltration of immune cells of samples in different risk stratifications in the TCGA dataset. (d) Infiltration of immune cells of samples in different risk stratifications in the CGGA dataset. ESTIMATE, Estimation of STromal and Immune cells in MAlignant Tumor tissues using Expression data.

**Figure:**
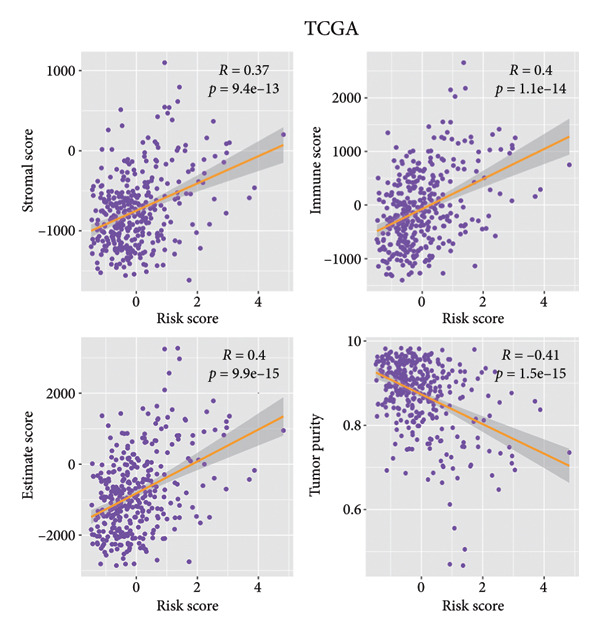
(a)

**Figure:**
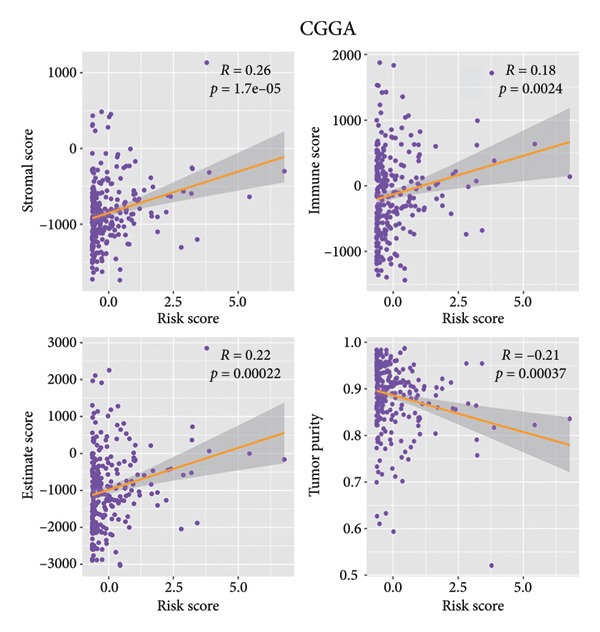
(b)

**Figure:**
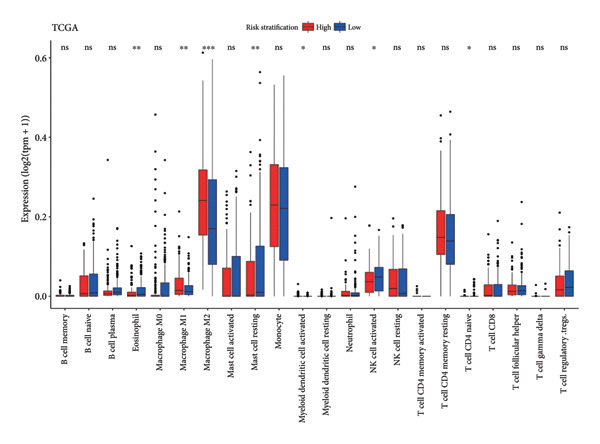
(c)

**Figure:**
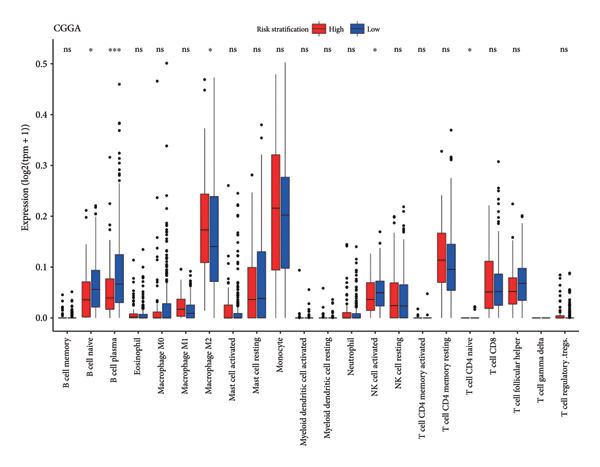
(d)

### 3.5. Connection Between the Efficacy of Immunotherapy and the Risk Score

Then the link between risk stratification and ICI effectiveness was investigated. In the TCGA dataset, the expression of multiple immune checkpoints in the high‐risk stratification was considerably greater than that in the low‐risk stratification (Figure 7(a)). With the increasing of patients’ risk scores, the TIDE scores (*R* = 0.15, *p* = 0.0056) and infiltration of cancer‐associated fibroblasts (CAF) (*R* = 0.45, *p* < 2.2 × 10^ (−16)) for individuals with LGG rose (Figure 7(b)). The higher expression of immune checkpoints, TIDE scores (*R* = 0.15, *p* = 0.0012), and infiltration of CAFs (*R* = 0.4, *p* = 6 × 10^ (−12)) were likewise connected with higher risk scores in the CGGA dataset (Figures 7(c) and 7(d)). This finding suggests that the high‐risk stratification had a greater chance of tumor immune evasion and that the efficacy of ICIs was worse in the high‐risk stratification. Individuals with high‐risk scores and high *PD-L1* expression showed a substantially grimmer prognosis than those with low‐risk scores. Additionally, individuals with low‐risk scores and high *PD-L1* expression had longer survival compared to those with high‐risk scores and high *PD-L1* expression (*p* < 0.0001) (Figure 7(e)). Patients with low‐risk scores appeared to have a better prognosis regardless of whether *PD-1* and *CTLA-4* were highly or poorly expressed (*p* < 0.0001) (Figures 7(f) and 7(g)). To investigate the correlation between the four‐CRL signatures and immunotherapy efficacy, we retrieved data from the IMvigor210 dataset, a urothelial carcinoma cohort treated with atezolizumab. The results showed that the four‐CRL signatures had a strong association with the survival outcomes of urothelial carcinoma patients receiving atezolizumab treatment (*p* = 0.034) (Figure 7(h)).

Figure 7Correlation between risk stratification and the efficacy of immunotherapy. (a) Expression of immune checkpoints between different risk stratifications in the TCGA dataset. (b) TIDE results of samples in the TCGA dataset. (c) Expression of immune checkpoints between different risk stratifications in the CGGA dataset. (d) TIDE results of samples in the CGGA dataset. (e and g) Kaplan–Meier analyses of overall survival among four patient groups stratified by the CRL signature and PD‐L1 (e), PD‐1 (f), and CTLA‐4 (g). (h) Kaplan–Meier analysis of urothelial carcinoma patients stratified by CRL signature in the IMvigor210 cohort. PD‐L1, programmed death‐ligand 1. PD‐1, programmed cell death protein 1. CTLA‐4, cytotoxic T‐lymphocyte antigen 4.

**Figure:**
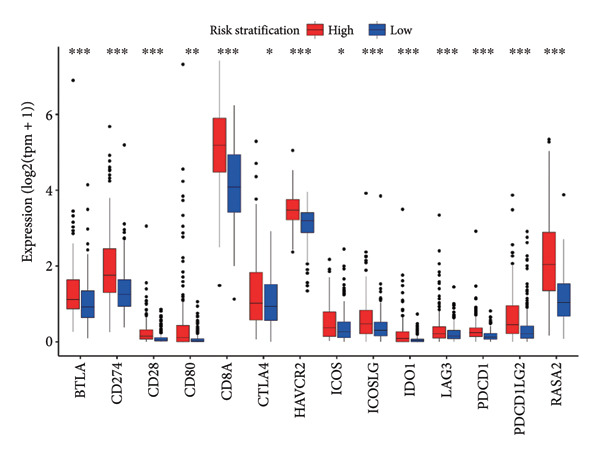
(a)

**Figure:**
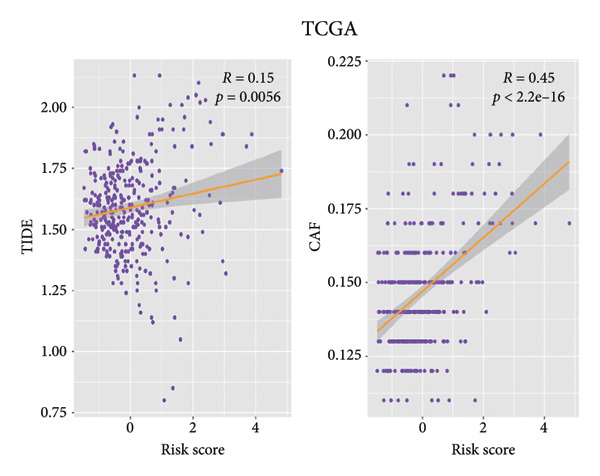
(b)

**Figure:**
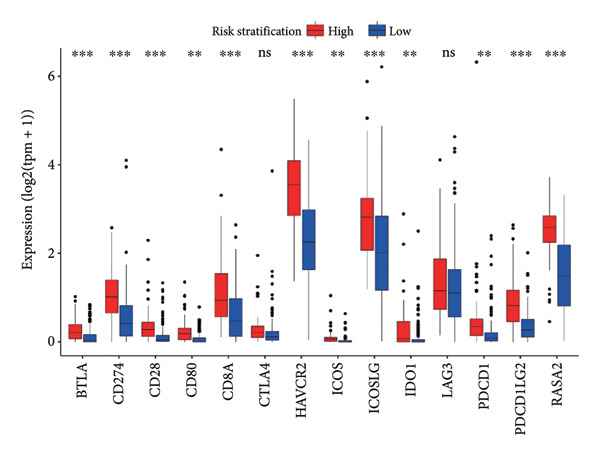
(c)

**Figure:**
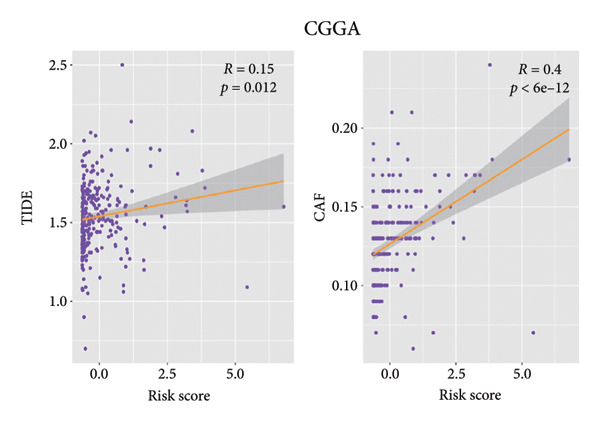
(d)

**Figure:**
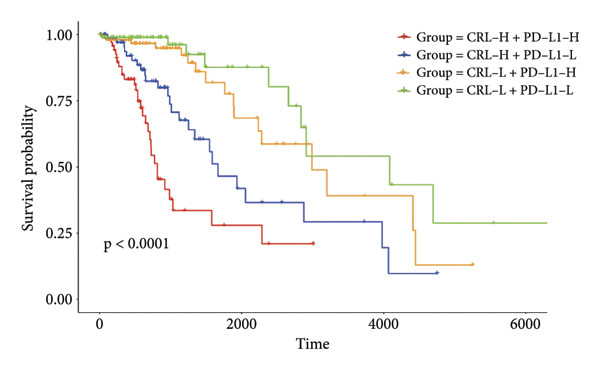
(e)

**Figure:**
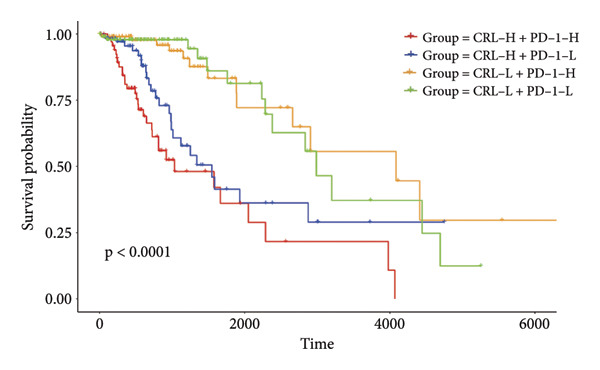
(f)

**Figure:**
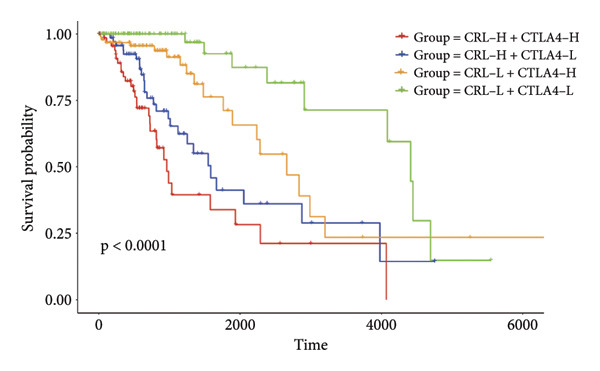
(g)

**Figure:**
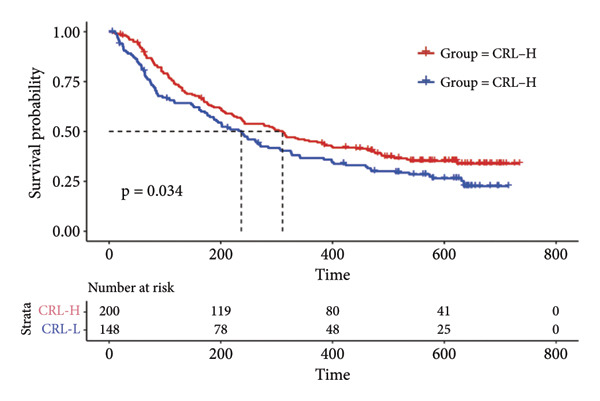
(h)

### 3.6. Enrichment Analysis of Molecular Mechanisms Underlying the Four‐CRL Signature

To assess functional pathway dynamics in LGG samples, we performed GSVA and GSEA between different risk stratification groups. GSEA revealed that apoptosis (*p* < 2.2 × 10^ (−16)), JAK–STAT pathway (*p* < 2.2 × 10^ (−16)), neutrophil extracellular trap formation (*p* < 2.2 × 10^ (−16)), the NOD‐like receptor pathway (*p* < 2.2 × 10^ (−16)), the PI3K–Akt pathway (*p* < 2.2 × 10^ (−16)), and the Rap1 pathway (*p* = 0.0075) were increased in the high‐risk stratification of TCGA (Figure 8(a)). Figure 8(b) showed the top 30 enriched GO terms in the high‐risk stratifications. The pathways identified as enriched in the TCGA dataset were likewise prominent in the high‐risk stratification of CGGA (Figure 8(c)). Figure 8(d) showed the top 30 enriched GO terms in the high‐risk stratifications. Similar to the GSEA results, the GSVA results showed that apoptosis, the JAK–STAT pathway, and the NOD‐like receptor pathway were considerably enriched in the high‐risk stratification group (Figures 8(e) and 8(g)). In addition, Figures 8(f) and 8(h) respectively showed the top 15 enriched GO terms in two stratifications.

Figure 8Bioinformatic analysis of molecular mechanisms between different risk stratifications. (a) GSEA results for KEGG pathways in the TCGA dataset. (b) GSEA results for GO terms in the TCGA dataset. (c) GSEA results for KEGG pathways in the CGGA dataset. (d) GSEA results for GO terms in the CGGA dataset. (e) GSVA results for KEGG pathways in the TCGA dataset. (f) GSVA results for GO terms in the TCGA dataset. (g) GSVA results for KEGG pathways in the CGGA dataset. (h) GSVA results for GO terms in the CGGA dataset. GSVA, gene set variation analysis. GSEA, gene set enrichment analysis. GO, gene ontology. KEGG, Kyoto Encyclopedia of Genes and Genomes.

**Figure:**
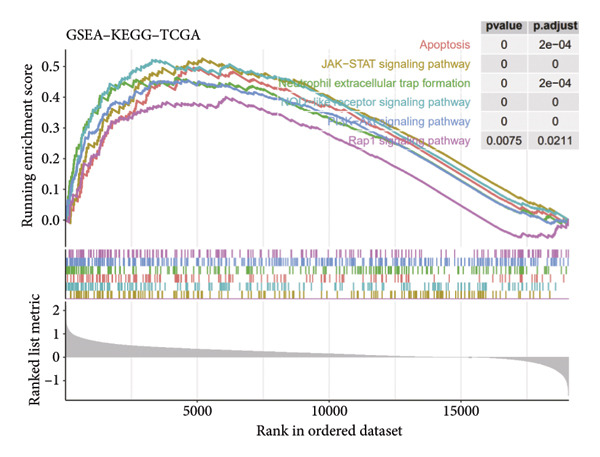
(a)

**Figure:**
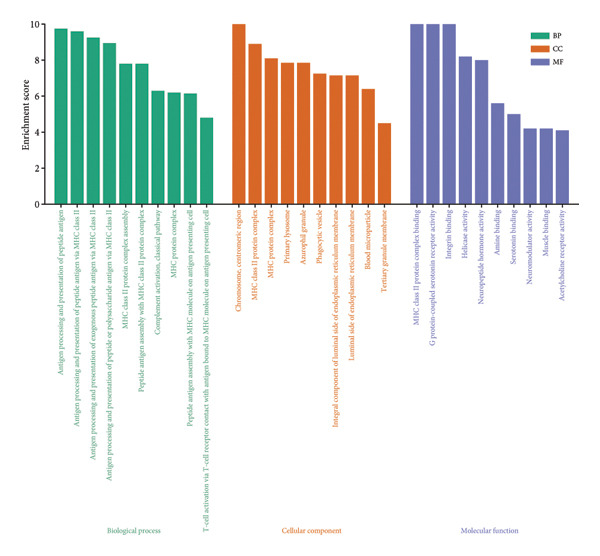
(b)

**Figure:**
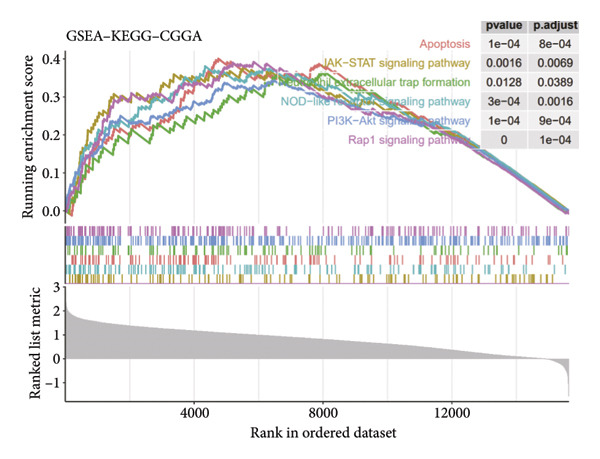
(c)

**Figure:**
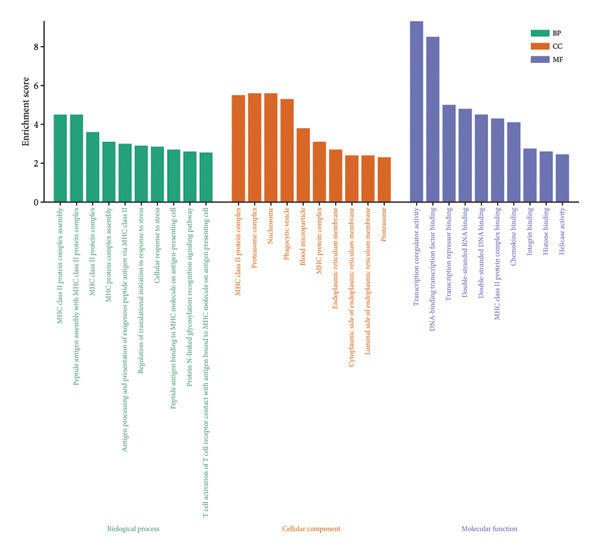
(d)

**Figure:**
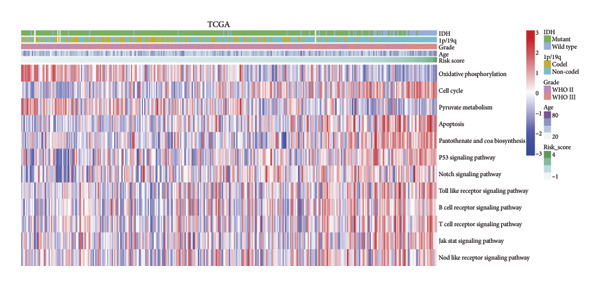
(e)

**Figure:**
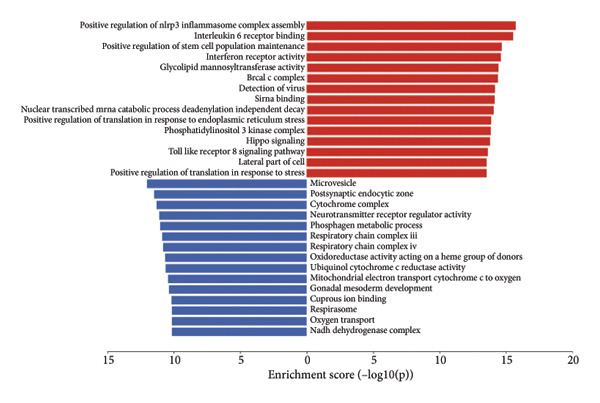
(f)

**Figure:**
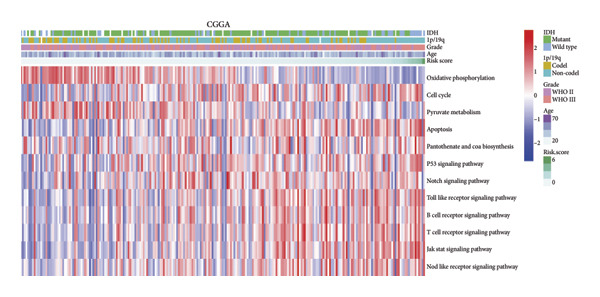
(g)

**Figure:**
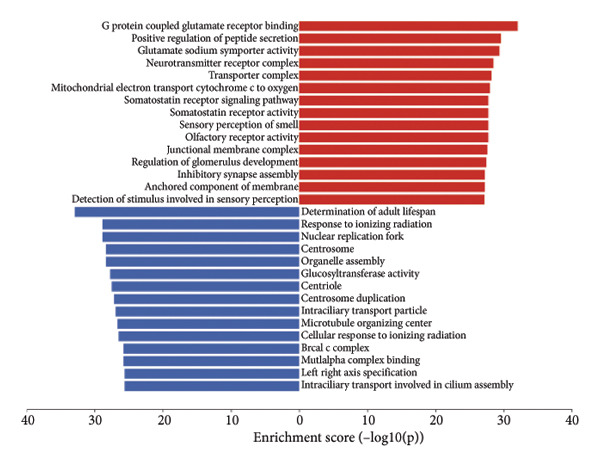
(h)

### 3.7. Prediction and Validation of Potential Targeted Drugs for Samples in High‐Risk Stratification Group

Based upon the expression and drug‐sensitivity data from the GDSC and CTRP v2 datasets, prospective sensitive pharmaceuticals for samples in different risk stratifications were predicted. Figure 9(a) showed the flowchart of predicting prospective pharmaceuticals. Samples from different TCGA risk groups showed distinct responses to 407 and 164 compounds from the CTRP v2 and GDSC2 datasets, respectively. In the CGGA cohort, samples from different risk groups showed distinct responses to 437 and 169 compounds from the CTRP v2 and GDSC2 datasets, respectively, and nine overlapped drugs were obtained for further analysis (Figure 9(b)). After the Spearman correlation analysis, five drugs (PLX‐4720, BMS‐536924, MG‐132, AZD8055, and AZD6482) were recognized as the prospective pharmaceuticals for samples in different risk stratifications, and The drug‐sensitivity patterns observed in CTRP v2 were consistent with those in GDSC2 across both TCGA and CGGA cohorts (Figures 9(c), 9(d), 9(e), 9(f)). Among these five potential drugs, four drugs (PLX‐4720, BMS‐536924, MG‐132, and AZD6482) were potential targeted drugs for samples in the high‐risk stratification, and AZD8055 could be more effective for samples in the low‐risk stratification.

Figure 9Prediction for potential targeted drugs for samples in the high‐risk stratifications. (a) Flow chart to predict potential drugs. (b) Venn diagram to filter out overlapped drugs in the GDSC2 and CTRP v2 datasets. (c) Sensitivity scores of drugs from the GDSC2 and CTRP v2 datasets in different risk stratifications in the TCGA dataset. (d) Correlation between risk scores and sensitivity scores of drugs from the GDSC2 and CTRP v2 datasets in the TCGA dataset. (e) Sensitivity scores of drugs from the GDSC2 and CTRP v2 datasets in different risk stratifications in the CGGA dataset. (f) Correlation between risk scores and sensitivity scores of drugs from the GDSC2 and CTRP v2 datasets in the CGGA dataset. GDSC, the genomics of drug sensitivity in cancer database. CTRP, the cancer therapeutics response portal. Sensitivity score, 50% inhibitory concentration.

**Figure:**
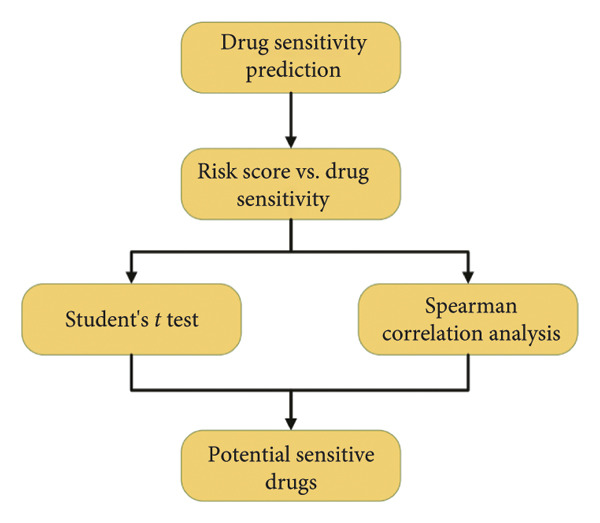
(a)

**Figure:**
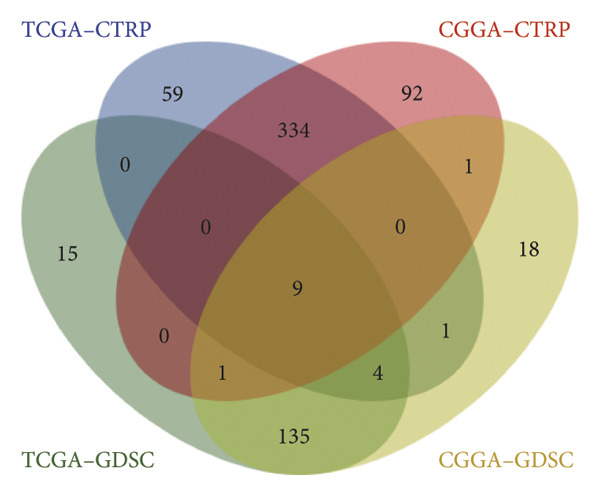
(b)

**Figure:**
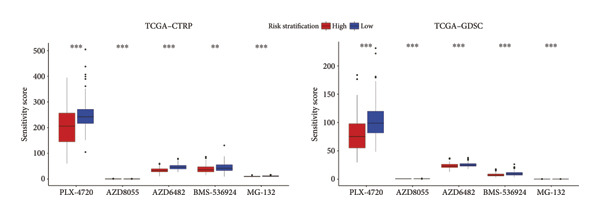
(c)

**Figure:**
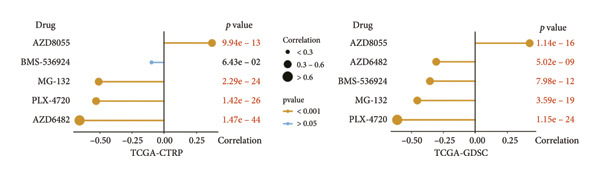
(d)

**Figure:**
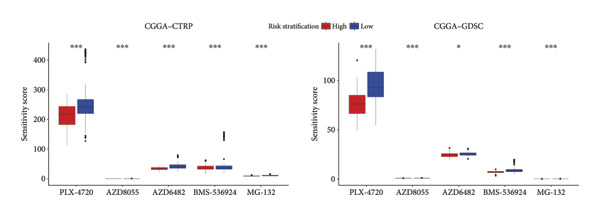
(e)

**Figure:**
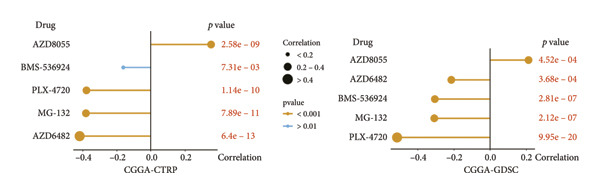
(f)

We performed several experiments to provide more evidence of the response of glioma to the predicted drug. We validated the effect of MG‐132, the drug with the lowest sensitivity score among the four prospective pharmaceuticals for individuals at high‐risk, to kill glioma cells. The colony formation and CCK8 experiments indicated that MG‐132 could considerably reduce the capacity to proliferate and form clones of glioma cells (Figures 10(a) and 10(b)). What is more, the findings of the wound healing experiment suggested that MG‐132 inhibits the migratory ability of glioma cells (Figure 10(c)). In summary, our results supported the accuracy of our drug prediction and revealed that MG‐132 was a strong antiglioma medication.

Figure 10Drug‐sensitivity experiment of predicted drug. (a) Colony formation assay for MG‐132. (b) CCK8 assay for MG‐132. (c) Wound healing assay for MG‐132.

**Figure:**
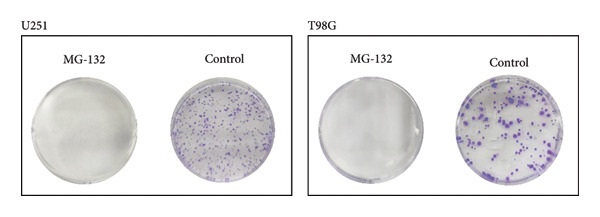
(a)

**Figure:**
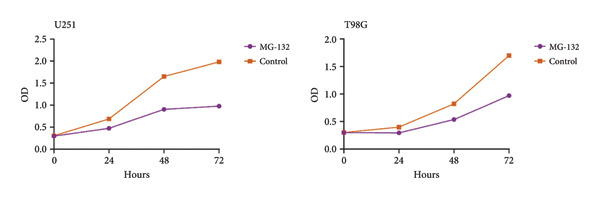
(b)

**Figure:**
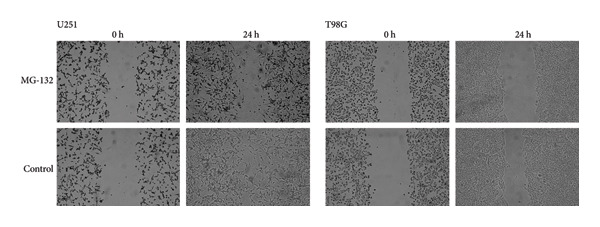
(c)

### 3.8. Experimental Validation of CRL‐Associated Malignant Phenotypes in Glioma

**Figure 11:**
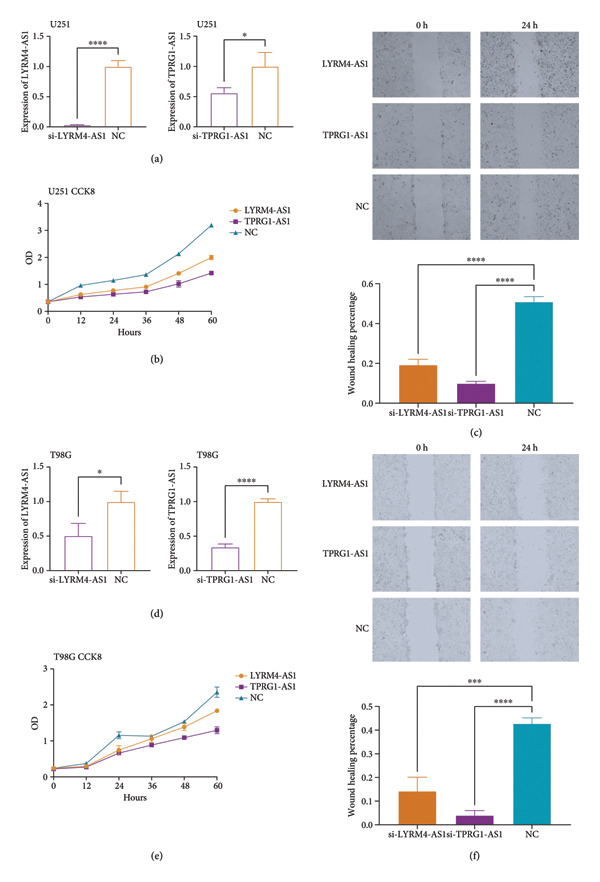
In vitro validation of the relationship between CRLs and glioma malignancy. (a) qPCR results showing the knockdown of LYRM4‐AS1 and TPRG1‐AS1 in U251 cells. (b) CCK8 assay showing the impact of knocking down two CRLs on the proliferation of U251 cells. (c) Wound healing assay showing the impact of knocking down two CRLs on the migration of U251 cells. (d–f) The corresponding qPCR, CCK8 assay, and wound healing assay results of the relationship between CRLs and glioma malignancy in T98G.

We selected and validated the role of *LYRM4-AS1* and *TPRG1-AS1* in the glioma malignancy, which were the two CRLs most linked with the prognosis of individuals with LGG. The transfection of siRNA‐*LYRM4-AS1* and siRNA‐*TPRG1-AS1* could effectively knock down the expression of *LYRM4-AS1* and *TPRG1-AS1*, respectively, in U251 cells (Figure 11(a)). The CCK8 experiment demonstrated that after the knockdown of the expression of the *LYRM4-AS1* (*p* < 0.0001) and *TPRG1-AS1* (*p* < 0.05), the proliferation capacity of the U251 cells was considerably suppressed (Figure 11(b)). The wound healing experiment demonstrated that the migratory capacity of the U251 cells was substantially suppressed after the knockdown of the expression of *LYRM4-AS1* (*p* < 0.0001) and *TPRG1-AS1* (*p* < 0.0001) (Figure 11(c)). These findings were consistent in another glioma cell line, T98G (Figures 11(d), 11(e), 11(f)). The knockdown of the expression of *LYRM4-AS1* (*p* < 0.001) and *TPRG1-AS1* (*p* < 0.0001) could effectively inhibit the proliferation and migratory capacities of T98G cells.

## 4. Discussion

CRLs were observed to be linked with the progression of glioma. Yan et al. originally examined the mechanism of CRLs in LGG and provided a few suggestions for novel treatment of LGG (including primary and recurrent) [22]. Wang et al. constructed a risk model underlying CRLs demonstrating strong prognostic prediction capacity while also reflecting the complicated microenvironment of glioma [21]. The results of the univariate Cox analysis showed that the high expression of cuproptosis‐related gene signatures, including *GCSH*, *ATP7B*, *FDX1*, *GLS*, *DLAT*, *MTF1*, *ATP7A*, and *SLC31A1*, was substantially linked with poor prognosis among individuals with LGG. This indicates that cuproptosis may be related to a grim outcome and malignant progression of LGG. Based on these eight prognostic cuproptosis‐related genes, 963 CRLs were identified. Subsequently, LASSO further screened four CRLs: *AC002456.1*, *TPRG1-AS1*, *AC098851.1*, and *LYRM4-AS1*. *AC098851.1* and *TPRG1-AS1* were correlated with the expression of *MTF1*, while *AC002456.1* was mainly associated with the expression of *FDX1*. Besides, the expression of *LYRM4-AS1* was associated with the expression of *DLAT*, *MTF1*, *ATP7A*, and *SLC31A1*.

Four CRLs, *AC002456.1*, *TPRG1-AS1*, *AC098851.1*, and *LYRM4-AS1*, constituted this novel risk signature. A previous study showed that *AC002456.1* is an immune‐related lncRNA closely related to the efficacy of immunotherapy on glioblastoma [38]. Furthermore, the results of Choi et al. demonstrated that *TPRG1-AS1* can inhibit the advancement of liver cancer through the competing endogenous RNA network [39]. This is the first study to reveal a strong association between *AC098851.1*, *LYRM4-AS1*, and LGG prognosis. By way of a series of in vitro experiments, we demonstrated a close relationship between two CRLs, *LYRM4-AS1* and *TPRG1-AS1*, and the migration and proliferation ability of glioma cells. *LYRM4* is present in the mitochondria and nucleus, where it binds to cysteine desulfurase and supports the release of inorganic sulfur from iron–sulfur clusters. Disruption of this gene negatively affects mitochondrial and cytoplasmic iron homeostasis [40–42]. One of the key characteristics of cuproptosis is iron–sulfur protein loss [19]. Therefore, LYRM4‐AS1 may potentially influence cuproptosis in LGG by regulating LYRM4, a gene involved in iron–sulfur cluster metabolism; however, this hypothesis requires further experimental validation and is currently under investigation in our laboratory.

The immune microenvironment was shown to contribute to the occurrence and development of gliomas [43, 44]. Studies have demonstrated that glioma cells could promote the formation of the immunosuppressive context surrounding gliomas; for example, glioma cells could stimulate tumor‐associated macrophages and regulatory T cells, further suppressing the functions of cytotoxic T cells [45]. What is more, the expression of immune checkpoints, for instance, *PD-L1,* was promoted to induce immune escape by glioma [46, 47]. Our findings demonstrated substantial variations in the immune microenvironment among individuals with LGG in different risk stratifications. Moreover, the findings of the immune infiltration analysis revealed that immunocyte infiltration was more significant in the high‐risk stratification, which was in keeping with the ESTIMATE findings. Studies have shown that high infiltration of M2 macrophages might enhance glioma growth and contribute to grim prognosis [48]. In our study, the infiltration of M2 macrophages in the high‐risk stratification was enormously increased in comparison to the low‐risk group, which might be linked to the grim prognosis for individuals with higher risk scores. Furthermore, high infiltration of NK cells can inhibit glioma’s progression in multiple ways [43, 49–51]. Our investigation demonstrated that the infiltration of NK cells in the high‐risk stratification was substantially reduced, which may contribute to the poor prognosis of patients with LGG. This suggests that cuproptosis might be connected to the imbalance of the immune microenvironment, especially that of M2 macrophages and NK cells.

Immunotherapy is a promising type of glioma treatment [45, 52, 53]. However, due to individual variability among patients, the response to ICIs varies among patients. Hence, establishing predictive biomarkers for ICI response will facilitate individualized treatments for glioma patients. The results of this study showed that the expression of multiple immune checkpoints in the high‐risk stratification was much greater than in the low‐risk group. The TIDE algorithm developed by Jiang et al. predicts the response to ICIs by quantifying the extent of dysfunctional T cells and infiltrating cytotoxic T lymphocytes, with higher TIDE scores indicating poorer tumor response to ICIs [28]. In the low‐risk stratification, both TIDE scores and immune checkpoint levels were markedly decreased, while patient survival was notably improved, suggesting that the risk model could serve as an indicator for evaluating the effectiveness of immunotherapy for individuals with LGG. Based on the TIDE results, the risk score had a positive correlation with the infiltration of CAFs, which is related to the immunosuppressive tumor microenvironment [54]. Additionally, lower risk scores may be associated with improved responses to ICIs. In the IMvigor210 cohort, the CRL signature was substantially appertained to the outcome of patients with urothelial carcinoma, with a high‐risk score indicating a positive outcome. Pursuant to our data, patients in the high‐risk stratification showed higher extent of immune infiltration and a positive response to immunotherapy, which might be the explanation of the positive outcome of patients with high‐risk scores in the IMvigor210 cohort. This result indicates that ICIs might be beneficial to individuals with LGG with strong expression of the four‐CRL signature.

It is reported that the PI3K/Akt pathway and Rap1 pathway are widely involved in the progression of glioma, including proliferation, invasion, and migration [55–57]. A study reported that IL‐8 recruited neutrophils, which subsequently triggered neutrophil extracellular trap formation via the PI3K/AKT/ROS axis to promote glioma progression [58]. The GSEA findings in our research revealed that the PI3K/Akt pathway, Rap1 pathway, and neutrophil extracellular trap formation were considerably enriched in the high‐risk stratification, which indicated that cuproptosis might regulate the progress of LGG via these pathways. For example, cuproptosis might crosstalk the PI3K/Akt pathway since the PI3K/Akt pathway was demonstrated to be activated by Cu^2+^ [59, 60], and this hypothesis needs to be further experimentally confirmed. What is more, the apoptosis pathway, JAK–STAT pathway, and NOD‐like receptor pathway were elevated in the high‐risk stratification by both GSEA and GSVA. Before cuproptosis was described, it was widely reported that copper is related to apoptosis and autophagy by inducing oxidative stress [61, 62], which could contribute to the enrichment of the apoptosis pathway in the high‐risk stratification. Excessive copper exposure was demonstrated to modulate signaling pathways, including the JAK–STAT pathway and the NOD‐like pathway [63]. Therefore, similar to the PI3K/Akt pathway, cuproptosis might crosstalk the JAK–STAT pathway and NOD‐like pathway by sharing the same trigger: Excessive copper exposure, which needs to be further validated in vitro and in vivo.

Chemotherapy is a promising therapy for the management of glioma [64–66]. Temozolomide (TMZ), a DNA alkylating drug, is a first‐line drug for treating malignant glioma. However, TMZ’s curative effect remains unsatisfactory for the selective permeation of the blood–brain barrier and the TMZ resistance of glioma [67, 68]. It is of tremendous clinical relevance to generate additional chemotherapeutic pharmaceuticals that can treat glioma. Zhu et al. developed an efficient and powerful transferrin/aptamer‐conjugated mesoporous ruthenium nanosystem to treat glioma by chemical photodynamic therapy [69]. Johanns et al. found that combined dabrafenib and trametinib was a safe and effective treatment for patients with high‐grade glioma with BRAF point mutations [70]. Our results showed that PLX‐4720, AZD6482, BMS‐536924, and MG‐132 might be potential drugs for treating individuals who have high‐risk scores because risk scores exhibited a positive correlation with the sensitivities of these drugs. Previous studies have shown the effectiveness of these drugs for treating glioma. PLX‐4720, a BRAF inhibitor, could reduce the growing capacity of glioma cells independent of BRAF mutation status [71]. AZD6482, a phosphoinositide‐3 kinase inhibitor, could effectively decrease the malignant phenotype and result in apoptosis of glioma cells [72]. BMS‐536924, an ATP‐competitive IGF‐1R/IR inhibitor, may significantly lower the survival rate of glioma cells regardless of TMZ resistance [73]. MG‐132, a proteasome inhibitor, could induce C6 glioma cell apoptosis via oxidative stress [74]. According to our results, MG‐132 was especially recommended to treat patients with high‐risk scores since the sensitivity score of MG‐132 was the lowest and had a negative correlation with the risk score.

The present research included several limitations. The validation of this study was underlain by the use of the TCGA‐LGG dataset to screen the expression of prognostic CRLs, and four CRLs (*AC002456.1*, *TPRG1-AS1*, *AC098851.1*, and *LYRM4-AS1*) showed excellent predictive potential. However, additional important genes with predictive values were not examined in this investigation. Given that the prognostic model was developed and verified using data from public repositories and in vitro assays, evidence from in vivo research is necessary to further corroborate the statistical evidence we have presented.

## 5. Conclusion

This study explored the possible molecular mechanisms of how CRL regulates the progress of LGG and constructed a unique CRL risk signature that could forecast the outcome for individuals with LGG and be associated with immune infiltration in LGG. Meanwhile. What is more, the CRL signature provides a new chemotherapy and immunotherapy strategy for patients with LGG in different risk stratifications. In conclusion, CRLs may be novel prognostic biomarkers for LGG.

## Ethics Statement

The authors have nothing to report.

## Consent

The authors have nothing to report.

## Disclosure

All authors approved the submitted version.

## Conflicts of Interest

The authors declare no conflicts of interest.

## Author Contributions

Mengyang Wang and Faliang Duan conceived, designed, and supervised the study. Ming Luo and Jianmei Yang drafted the manuscript. Jingyi Yang and Lei Shen collected the data. Lei Shen performed data analysis and visualization. Mengyang Wang and Ming Luo performed validation experiments. All authors contributed to the article. Mengyang Wang and Jianmei Yang contributed equally to the work.

## Funding

This study did not receive any funding.

## Supporting Information

Supporting 1: Results of Spearman correlation analysis to identify 963 CRLs.

## Supporting information


**Supporting Information** Additional supporting information can be found online in the Supporting Information section.

## Data Availability

The TCGA‐LGG dataset for this study can be found in the GDC database (https://portal.gdc.cancer.gov/). The CGGA‐LGG dataset for this study can be found in the CGGA database (https://www.cgga.org.cn/). The IMvigor210 dataset for this study can be found in the R package IMvigor210CoreBiologies (https://research-pub.gene.com/IMvigor210CoreBiologies/). The GDSC2 dataset can be found in the GDSC database (https://www.cancerrxgene.org/). The CTRP v2 dataset can be found in the CTRP database (https://portals.broadinstitute.org/ctrp/).
